# Myocardial Viability Testing in the Management of Ischemic Heart Failure

**DOI:** 10.3390/life12111760

**Published:** 2022-11-01

**Authors:** Elena Emilia Babes, Delia Mirela Tit, Alexa Florina Bungau, Cristiana Bustea, Marius Rus, Simona Gabriela Bungau, Victor Vlad Babes

**Affiliations:** 1 Department of Medical Disciplines, Faculty of Medicine and Pharmacy, University of Oradea, 410073 Oradea, Romania; 2 Department of Pharmacy, Faculty of Medicine and Pharmacy, University of Oradea, 410028 Oradea, Romania; 3 Doctoral School of Biomedical Sciences, University of Oradea, 410087 Oradea, Romania; 4 Department of Preclinical Disciplines, Faculty of Medicine and Pharmacy, University of Oradea, 410073 Oradea, Romania

**Keywords:** coronary artery disease, heart failure, ischemic cardiomyopathy, left ventricular function, myocardial viability

## Abstract

Although major advances have occurred lately in medical therapy, ischemic heart failure remains an important cause of death and disability. Viable myocardium represents a cause of reversible ischemic left ventricular dysfunction. Coronary revascularization may improve left ventricular function and prognosis in patients with viable myocardium. Although patients with impaired left ventricular function and multi-vessel coronary artery disease benefit the most from revascularization, they are at high risk of complications related to revascularization procedure. An important element in selecting the patients for myocardial revascularization is the presence of the viable myocardium. Multiple imaging modalities can assess myocardial viability and predict functional improvement after revascularization, with dobutamine stress echocardiography, nuclear imaging tests and magnetic resonance imaging being the most frequently used. However, the role of myocardial viability testing in the management of patients with ischemic heart failure is still controversial due to the failure of randomized controlled trials of revascularization to reveal clear benefits of viability testing. This review summarizes the current knowledge regarding the concept of viable myocardium, depicts the role and tools for viability testing, discusses the research involving this topic and the controversies related to the utility of myocardial viability testing and provides a patient-centered approach for clinical practice.

## 1. Introduction

Ischemic heart disease is still the leading cause of morbidity and mortality. There is an increasing number of patients with ischemic cardiomyopathy and heart failure. Coronary artery disease (CAD) is the most important cause of chronic heart failure accounting for two thirds of left ventricular (LV) dysfunction cases [1]. Around 126 million individuals worldwide are suffering from ischemic heart failure [2]. 

LV function is one of the most important prognostic determinants in patients with CAD, impacting survival, hospitalization and quality of life [3]. Long-term survival is poor in patients with CAD and severe LV impairment. Strategies to identify those patients who will benefit from revascularization are warranted. Improving of symptoms, LV ejection fraction and increased overall survival were reported after the revascularization of dysfunctional but viable myocardium in observational studies. However, the peri-operative risk is significant, and revascularization may be quite challenging in this type of patients with poor ejection fraction [4,5,6,7,8]. Commonly these patients are old, frail and with multiple comorbidities [9]. Another challenge is represented by the coronary anatomy in patients with ischemic heart failure in whom multi-vessel complex CAD is frequent, and the procedural risk of myocardial revascularization is increased. The operative mortality for coronary artery by-pass graft (CABG) is significantly higher in patients with LV dysfunction [10]. The procedural risk is lower for percutaneous coronary intervention (PCI), but sometimes incomplete revascularization can diminish the benefits, and until now evidence for interventional revascularization in ischemic heart failure has been lacking [11].

**Figure 1:**
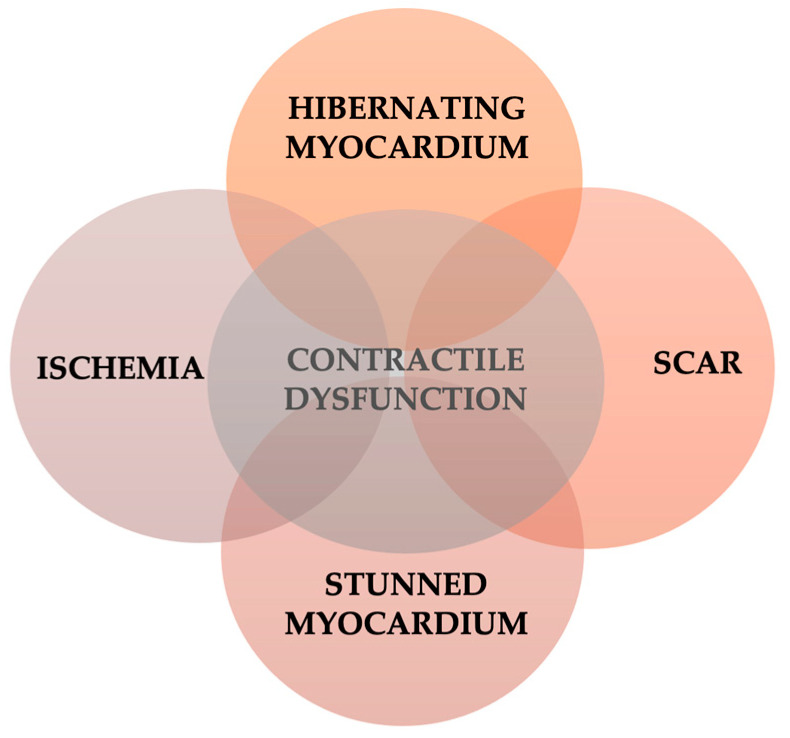
Dysfunctional myocardium in ischemic cardiomyopathy.

The improved survival after acute coronary syndromes has led to an increased prevalence of ischemic heart failure. The major priority in patients with acute coronary syndromes is to reduce the amount of myocardial necrosis with reperfusion strategies. However, despite early reperfusion, patients are prone to develop myocardial structural alterations that will reduce LV function and will lead to heart failure [3]. Around one third of the patients with acute myocardial infarction will develop heart failure [12,13,14,15]. LV remodeling after myocardial infarction and the consequent hemodynamic changes are major contributors to chronic heart failure. After an ischemic event, different processes can lead to impaired LV function. While cell death via necrosis or apoptosis is irreversible, other processes are to some extent reversible with appropriate therapy. In the setting of viable myocardium, the restoration of an adequate blood flow theoretically will restore myocardial contractility and will improve LV ejection fraction. In ischemic cardiomyopathy, the myocardium with contractile dysfunction encompasses a continuum from stunned to hibernating myocardium and necrosis with scar formation [16] (Figure 1).

Although observational studies reported that in patients with myocardial viability, outcomes improved following surgical revascularization, prospective randomized trials have not confirmed the positive interaction between myocardial viability and the effect of revascularization. The recovery of LV function after revascularization is a marker of myocardial viability but is not the most important element, and it does not seem to impact outcomes. Protection of the residual viable myocardium from subsequent acute coronary events and prevention of further deterioration are probably major contributors to improving outcome [17]. However, the results of this randomized research raised the question about the utility of myocardial viability testing before revascularization. It is also unclear if patients without a certain amount of viable myocardium on noninvasive testing have no benefits after surgical revascularization.

The role of viability testing in ischemic cardiomyopathy remains controversial, and there are still many unknown elements regarding the interaction between viability, ischemia and treatment in predicting clinical outcomes and prognosis [18].

The aim of this review is to provide a synthesized overview of the current knowledge regarding the concept of viable myocardium and the methodology of myocardial viability testing, to discuss controversies regarding usefulness of viability testing in the management of ischemic heart failure and to describe a potential approach for clinical practice.

## 2. Material and Methods

**Figure 2:**
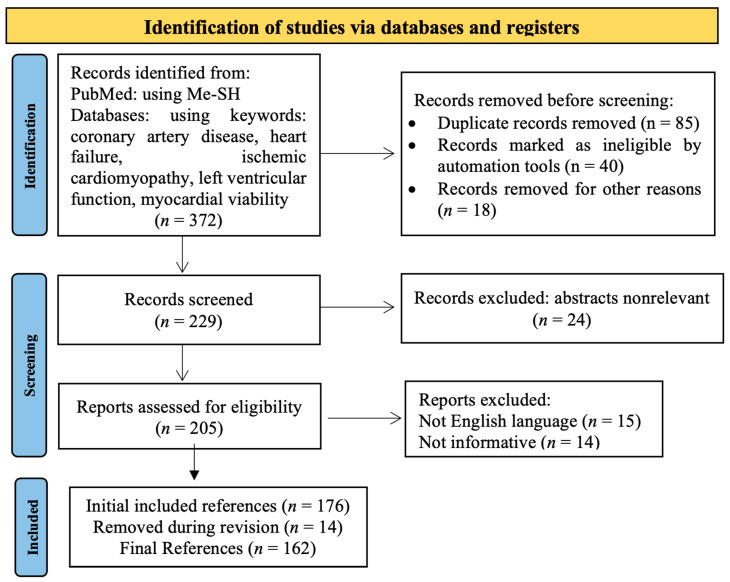
PRISMA flowchart describing the criteria of literature selection.

Extensive research of the literature was performed comprising scientific data regarding myocardial viability, from the concept of viable myocardium to the most important methods of assessing myocardial viability; observational studies and randomized clinical trials on this topic; the prognosis related to myocardial viability; and approaches in the management of patients with ischemic heart failure. Databases such as PubMed, Cochrane Library, Scopus, Web of Science, etc., were researched using keywords and Me-SH terms. The methodology of literature selection is presented in the PRISMA flowchart [19] (Figure 2). 

## 3. Viable Myocardium: Stunned and Hibernating

Following an acute ischemic event with abrupt interruption of the blood supply, myocardial necrosis was observed 30 min after ligaturing of the left anterior descending coronary artery in rats [20]. After the ischemic injury, multiple inflammatory mechanisms and metalloproteinases are activated producing the replacement of necrotic tissue with a fibrotic scar. The myocardium may remain viable if the severity and the duration of blood flow reduction is limited and/or the blood flow is restored, as in hibernating or stunned myocardium [12]. 

Myocardial stunning is a condition defined by reversible LV contractile dysfunction in the presence of a restored coronary flow after a brief period of coronary occlusion. In stunned myocardium, there is a flow–contraction mismatch with restored blood flow but persistent impaired contractility. The recovery of contractile dysfunction occurs spontaneously in a few hours or weeks [21]. 

The most common settings with stunning myocardium in humans are post-reperfusion after acute myocardial infarction, after cardioplegic arrest during open heart surgery and after exercise-induced ischemia or after coronary artery spasm. Various factors are involved in the pathophysiology of stunned myocardium, such as: oxygen free radicals and/or calcium overload and structural changes of collagen [1].

Hibernating myocardium is defined as a dysfunctional but viable cardiac area with persistently reduced contractility due to reduced coronary blood flow at rest. Myocardial contraction requires a higher amount of energy than that needed for cell survival. Myocardial cells are entering into a state of hibernation, where the energy is used to preserve cellular integrity to the detriment of contractile function [22,23]. This impairment of myocardial function is associated with an important reduction in myocardial energetics and metabolism, concomitantly with the reduction of coronary blood flow [3]. 

Rahimtoola was the first author to describe a significant recovery of myocardial function after revascularization and introduced this concept of viability [22]. Hibernating myocardium is a term that applies to segments that regain contractility after revascularization, but in clinical practice it is commonly used for the definition of viable myocardium prior to revascularization [24]. 

Coronary blood flow at rest is not so severely impaired as the degree of cardiac dysfunction, but rather coronary flow reserve is diminished. Hibernating myocardium may also result from repetitive stunning with progression over time from normal coronary flow with reduced flow reserve to reduced resting coronary flow [25]. The chronically reduced myocardial blood flow and coronary flow reserve will determine a reduced supply with oxygen and nutrients, the reduction of adenosine triphosphate (ATP) levels and an increased shift toward anaerobic glycolysis that will prevent cardiomyocyte necrosis. This state of low metabolic function is accompanied by structural changes: myocardial cell dedifferentiation, a reduction of myofilaments and of cytoskeleton proteins, degeneration of mitochondria and loss of sarcomeres and sarcoplasmic reticulum. This will result in the shrinking and atrophy of cardiomyocytes and activation of repairing mechanisms, which can promote interstitial fibrosis. After revascularization, redifferentiation and proliferation of myocardial cells are restored [26,27], and an early revascularization can prevent irreversible morphological alterations [28]. 

**Figure 3:**
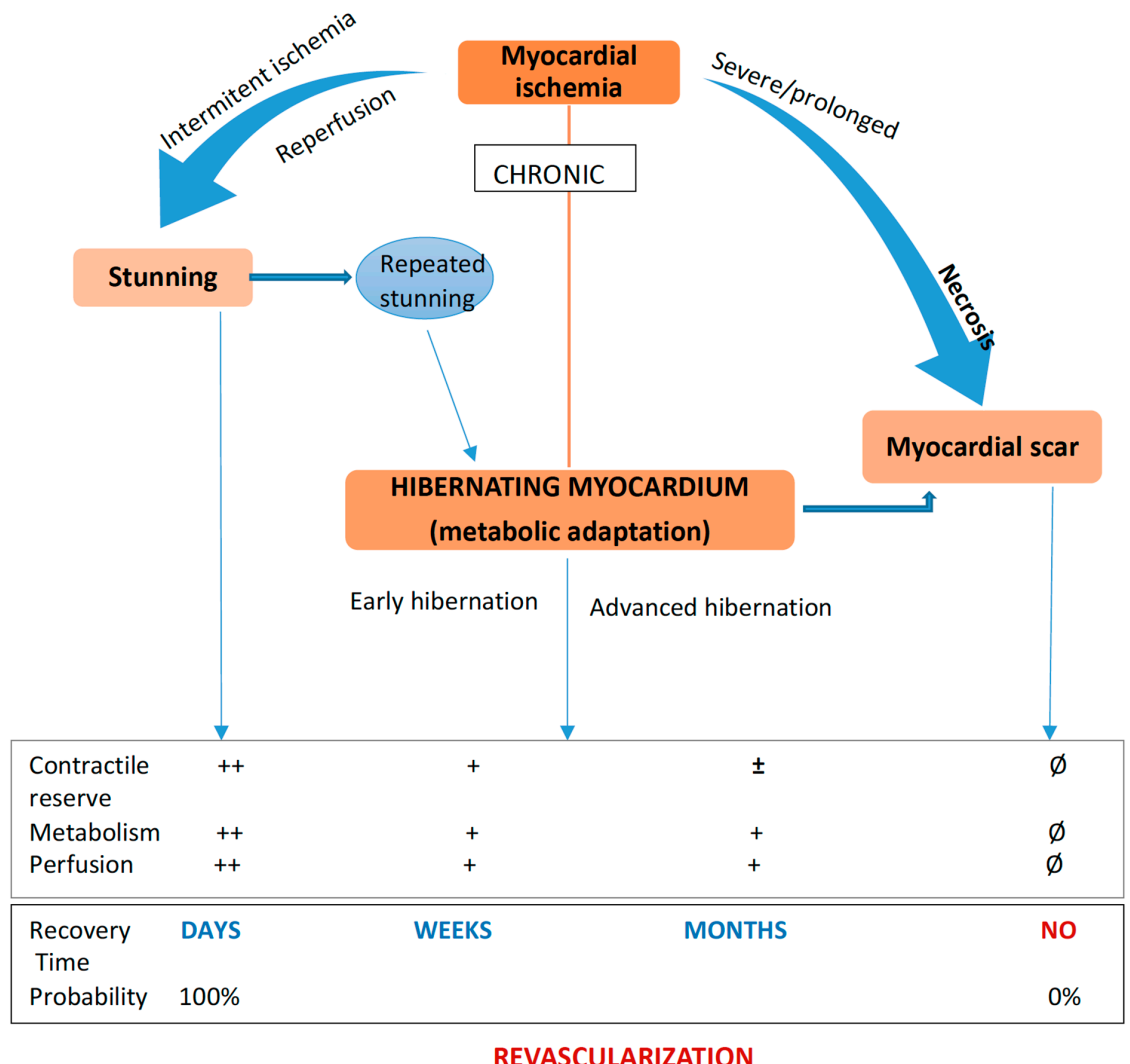
Pathophysiology of viable myocardium and probability of myocardial functional recovery.

Although they are described as different entities, this two-setting, stunned and hibernating myocardium can represent different stages of a continuum of LV dysfunction, and chronic, repetitive stunning can lead to myocardial hibernation [3]. The revascularization of hibernating myocardium can improve contractility, LV systolic function, morbidity and mortality. In a chronic state of ischemia, without adequate treatment, hibernating myocardium may be replaced by fibrosis as a continuum process [4,5]. The outcome of patients depends on the stage at which therapeutical intervention is made [29] (Figure 3).

## 4. Methods for Myocardial Viability Assessment

In clinical practice, myocardium with contractile dysfunction that recovers contractility with appropriate medical therapy and revascularization is a viable myocardium. An assessment of myocardial viability can be difficult, as viability is not a dichotomous concept, but rather there are different degrees of viability that can vary from total absence of viability to normality [30]. 

### 4.1. Markers of Myocardial Viability and Methods of Assessment

Hibernating myocardium is viable, so in different investigation methods, a conserved cell membrane integrity and metabolic activity, the absence of scar and the presence of contractile reserve can be identified [6]. Available imaging tools that assess myocardial viability can evaluate the structural and functional integrity of cardiomyocyte and the intracellular processes in the myocardial cells [31]. 

The preservation of LV wall thickness can be evaluated with echocardiography and cardiac magnetic resonance imaging (MRI), while the contractile reserve is investigated with dobutamine stress echocardiography (DSE) and stress cardiac MRI. 

Myocyte membrane integrity can be evaluated with single-photon emission computed tomography (SPECT) based on radiotracer uptake, and blood perfusion is evaluated with scintigraphy with Thallium (Tha) or technetium (Tc) contrasts, positron emission tomography (PET) and contrast cardiac MRI. 

PET and cardiac MRI spectroscopy may evaluate metabolic defects of non-viable myocardium. Late gadolinium hyper-enhancement (LGE) and T1 mapping cardiac MRI can depict myocardial fibrosis and scar. Diagnostic technique should have a good spatial resolution since necrotic myocardium and viable myocardium may coexist in the same segment of LV wall. Cardiac MRI is, from that point of view, the imaging diagnostic tool of choice.

The traditional definition of viable myocardium implies the recovery of myocardial function after revascularization. As a consequence, imaging tools that evaluate reserve contractility as DSE and stress cardiac MRI will possess a higher positive predictive value and specificity, but lower sensitivity in identifying viable myocardium, than methods that are evaluating cell membrane integrity such as SPECT, or metabolic activity such as PET, which have higher sensitivity and negative predictive value but a lower specificity [32]. 

A meta-analysis of 158 studies confirmed that PET had the highest sensitivity and negative predictive values, and DSE the highest specificity and positive predictive values at a segmental level [33]. 

### 4.2. Methods for Myocardial Viability Assessment

#### 4.2.1. Echocardiography

Echocardiography represents an available and inexpensive diagnostic tool able to provide information on myocardial viability. 

At rest, echocardiography can establish LV ejection fraction (LVEF), can detect the presence of wall motion abnormalities and allows measurements of wall thickness and LV size. Myocardial cell necrosis and fibrosis are leading to a thinning of the LV wall. Echocardiography can evaluate the thinning of the LV in akinetic or dyskinetic segments, and an increased refraction may indicate fibrosis. The presence of end-diastolic thickness at rest below 5 mm associated with akinesia or dyskinesia is a sign of non-viable myocardium [34,35].

A myocardial wall thickness over 5 mm in end-diastole is an indicator of a viable myocardium with a sensitivity of 100% but with a low specificity of 28%. A severely dilated LV can reflect the presence of non-viable myocardium, an increased end-systolic volume being correlated with poor LV function improvement after revascularization. An LV end-systolic volume > 130 mL can predict a higher rate of cardiac events after revascularization even in the presence of metabolically viable myocardium [24,36]. Echocardiography is largely available but has poor specificity in identifying viable myocardium. 

DSE has an established role for the evaluation of hibernating and stunned myocardium and can help in identifying those patients with ischemic cardiomyopathy that are suitable for revascularization. DSE has low risk, and is a quick and non-invasive investigation that implies no radiation, so is the most widely used technique for detecting myocardial viability [37]. 

DSE can evaluate the contractile reserve in the viable myocardium and predict functional recovery after revascularization. However, even in the absence of contractile reserve, myocardial viability may exist. In patients with a low amount of viable myocardium, revascularization may not be followed by contractility recovery, but LV remodeling can be avoided [30]. Dobutamine at low doses between 5 and 10 µg/kg/min will stimulate a contractile response in viable segments of the myocardium. The presence of a contractile reserve in a minimum of 5 segments is a good predictor of LV function improvement after revascularization. At higher doses, an increase in oxygen consumption can produce ischemia in territories deserved by stenotic coronary arteries [38]. 

An initial improvement of contractility indicating viability, followed by a later worsening due to ischemia, is a biphasic response characteristic for hibernating myocardium. A biphasic response has a reported sensitivity of 60% and a specificity of 88% in predicting the recovery of contractile function after revascularization [37]. The second most characteristic response to dobutamine for hibernating myocardium is the worsening of contractile function as the dobutamine dose is increasing. A partial viable myocardium may have no contractile reserve and DSE may further worsen contractility. The combination of a biphasic response with a worsening response improves sensitivity to 74% but decreases specificity to 73% in prediction of myocardial contractility recovery [39,40]. When a sustained improvement in contractility is observed, with a progressive increase in the dobutamine dose, a normal restored blood flow is probably present (stunned myocardium), in the area of a non-transmural or remodeled infarction, or it is a normal response in non-ischemic dilated cardiomyopathy [41]. If the myocardium is scarred and non-viable, no contractile response will develop during DSE [42], but this finding is not very sensitive in the detection of non-viable myocardium. The absence of the contractile reserve does not mean the absence of viability, especially in advanced stages with thinned hibernating myocardium. Improved detection of myocardial borders can be achieved with harmonic imaging, contrast echocardiography and trans-esophageal echocardiography [43,44].

Compared to SPECT and PET, DSE has a greater specificity (81% vs. 73%) but a lower sensitivity (84% vs. 90%) for the detection of myocardial viability [12,45,46].

Myocardial contrast echocardiography can assess microvascular integrity with a contrast agent, and perfusion defects are indicators of the no-reflow phenomenon associated with remodeling and poor outcome [47]. Microvascular integrity is a determinant of maximal contrast intensity, and the preservation of microvascular integrity and perfusion predicts the recovery of dysfunctional ischemic myocardium. Myocardial segments are visually classified as being viable, with normal or patchy perfusion, and non-viable when perfusion is absent [48]. Furthermore, myocardial contrast echocardiography allows a better evaluation of the LV borders [49]. The reported sensitivity of the method is 62–96%, but the specificity is low between 18 and 67%. DSE has a greater specificity versus contrast echocardiography for detecting myocardial viability [50,51].

Korosoglou et al. performed a study to compare myocardial contrast echocardiography with DSE and with combined Tc 99m SPECT and FDG-18 PET for the evaluation of myocardial viability. Myocardial contrast echocardiography showed 86% sensitivity and 43% specificity to predict myocardial function recovery, nuclear imaging had 90% sensitivity and 44% specificity and DSE had 83% sensitivity and 76% specificity [50]. Myocardial contrast echocardiography combined with dobutamine stress can better predict myocardial function recovery with an increased sensitivity of 96% and 63% specificity [38].

An accurate test for the evaluation of regional myocardial deformation is speckle-tracking echocardiography [24]. By evaluating the strain, respectively, the deformation of the myocardium and the strain rate, respectively, the gradient of the velocities of this deformation, speckle-tracking echocardiography can detect subtle wall motion abnormalities. Longitudinal strain can differentiate between viable and non-viable myocardium with a sensitivity of 81%, and a reduction in circumferential strain is a strong predictor for the absence of viability [52].

#### 4.2.2. Gated Single-Photon Emission Computerized Tomography (SPECT)

The SPECT permits the concomitant evaluation of perfusion and contractility at both global and segmental levels. The sensitivity of the method is 87% and specificity 65% in predicting the recovery of contractility after revascularization [12,53].

In SPECT imaging, a viable myocardium is characterized by the presence of cardiomyocyte with cellular membrane integrity that uptakes a radionuclide-labeled tracer. An uptake of >50% tracer activity of the maximum uptake of a normal segment is used to identify viable myocardium, and an uptake less than 30% is indicative of non-viable myocardium. in an uptake between 30 and 50% of tracer activity, a partial viable myocardium is identified. 

The most commonly used radiotracers in SPECT myocardial perfusion imaging are Thallium 201 and Technetium 99m. When rest perfusions’ defects are detected, and the viability of myocardium is questionable, stress perfusion imaging may be performed. A worsening defect on stress imaging (indicating ischemia) can suggest viable myocardium. A preserved wall motion and thickening of the myocardium at rest, or improving of segmental contractility on post-stress gated imaging is an important marker of the absence of a transmural scar [12]. 

Thallium 201 is a cationic radionuclide isotope analog of potassium. The initial myocardial uptake of Thallium 201 is determined by rest coronary blood flow, but the subsequent uptake in the next 4–24 h is produced by ”refill and redistribution” of the tracer, depending on cellular membrane integrity [32,41]. the intracellular uptake of Thallium 201 is produced by a passive diffusion mechanism but also by an active mechanism through the Na/K-ATP-ase pump. In myocardial areas with normal blood flow, the uptake is fast and high concentrations are rapidly obtained, and the speed of uptake is proportional to the coronary flow. In areas with impaired flow, tissue dedifferentiation or both, the uptake of Thallium is slower and final concentrations are lower. After uptake in the myocardial cells, redistribution is initiated which consists in a cation exchange between the cells and the blood with a duration that depends on the regional coronary blood flow. Reinjection of a small dose of Thallium will increase redistribution and will provide a better contrast between areas with a normal or abnormal uptake. Late redistribution and redistribution reinjection are both very sensitive for detecting myocardial viability [54,55]. 

Hibernating myocardium will appear as a perfusion defect, determined by reduced rest coronary blood flow on initial images, but in 4–24 h imaging, the uptake will become normal due to the redistribution of Thallium 201 (with or without reinjection). The sensitivity in detecting viable myocardium may increase in the late 24 h reinjection/redistribution protocol versus 4 h early redistribution protocol [12].

A quarter of fixed defects on reinjection Images are reversible on nitrate/reinjection images, improving the identification of myocardial viability [55]. 

Technetium (Tc) 99m compounds have a greater photonic energy and lower half-life than Thallium, being better detected by SPECT gamma cameras. Tc 99m-sestamibi and Tc 99m-tetrofosmin are lipophilic with cardiac affinity and are distributed in the myocardial cells proportional with coronary flow. Technetium enters in the myocardial cells through passive diffusion via the cell and mitochondrial membrane. Its fixation is more than 90% in the mitochondria and depends on the presence of the mitochondrial metabolic activity and maintenance of the polarity of the transmembrane potential. Redistribution is not produced, and Tc remains inside the cell. 

Technetium compounds are very good in the evaluation of coronary blood flow because no redistribution occurs, but it is impossible to differentiate between hypo fixation due to reduced coronary flow or cellular necrosis. It is essential to measure relative levels of activity in different myocardial regions, with Tc 99m-sestamibi activity being inversely correlated with the amount of fibrosis. Activity cutoff points above 50–55% predicted the functional recovery of myocardium after revascularization [56,57].

A perfusion defect at rest is myo”ardi’l infarction or hibernating myocardium. If the perfusion defect is correlated with the presence of wall motion and thickening on gated imaging (rest or post-stress), and there is a worsening of perfusion defect at stress, this indicates a hibernating myocardium. 

Although Thallium is considered superior in myocardial viability assessment due to its redistribution, different studies showed similar sensitivity and specificity in detecting viable myocardium for both tracers and in predicting functional recovery after revascularization (86% and 59% for Thallium, 81% and 66% for Tc 99m [32,41]). 

SPECT is more available and cheaper than PET, but the absence of tracer uptake does not confirm non-viable myocardium, while the normal uptake of radiotracer confirms viability. A thinned viable myocardium can sometimes be characterized on SPECT imaging as non-viable due to poor resolution, and DSE may detect the absence of contractile reserve in thinned non-fibrotic myocardial segments mislabeling a viable or partially viable myocardium as non-viable [41].

The low spatial resolution Is affecting the accuracy of diagnosis. SPECT can detect about half of non-viable segments with involvement of less than 50% of wall thickness detected by LGE cardiac MRI [58]. New isotopes are under research as: Tc 99m-nitrindo complexes (DBODC5) and mitochondrial complex-1 inhibitors as I 123-CMICE-013, but further studies are required [59].

#### 4.2.3. Positron Emission Tomography (PET)

PET implies the comparison of myocardial perfusion and myocardial metabolism to assess myocardial viability [60]. The main positron emitters for detecting myocardial viability are F-fluorodeoxyglucose (FDG) and 13N-ammonia or Rubidium-82 (Rb 82). FDG detects myocardial glucose metabolism, and 13N-ammonia or Rb 82 can assess coronary blood flow. An increased glucose metabolism coupled with reduced coronary blood flow is present in the dysfunctional ischemic viable myocardium. However, it is sufficient to analyze the glucose metabolism in myocardial cells to evaluate myocardial viability. A healthy myocardium is using free fatty acids to produce ATP, but in an ischemic hibernating myocardium there is a shift of metabolism from free fatty acids to anaerobic glycolysis. For the optimal uptake of FDG by the viable myocardium, it is important to have adequate patient preparation, with the stimulation of endogenous insulin release. The optimal preparation of diabetic patients is sometimes difficult and requires insulin injection. Rest PET imaging with perfusion tracer and metabolic tracer FDG are used for viability assessment. A normal rest perfusion confirms viable myocardium, and there is no need for FDG testing. A mismatch pattern of reduced perfusion at rest with a preserved FDG uptake is characteristic for viable myocardium [61]. Absent myocardial perfusion and absent FDG uptake are considered a non-viable transmural scar. Partially reduced myocardial perfusion and concordant FDG uptake reflect a non-transmural scar [12].

FDG imaging can detect myocardial viability with a high sensitivity of 92%, a lower specificity of 63%, a positive predictive value of 74% and a negative predictive value of 87% [12,53].

The main disadvantages are low availability and high cost, the complex and long protocol, interference of FDG uptake by medication (levothyroxine), diabetes, age, sex, heart failure and inability to identify small subendocardial necrosis. Advantages of PET over SPECT are considered as follows: superior spatial resolution, less radiation exposure and quick and absolute quantification of myocardial perfusion. A superior diagnostic accuracy is revealed for PET in the detection of myocardial viability versus other imaging tools [41].

#### 4.2.4. Cardiac Magnetic Resonance Imaging (MRI)

Cardiac MRI can evaluate myocardial viability with various techniques that will assess the metabolic, structural, functional alterations, tissue characteristics and cellular viability. It is the ideal imaging technique for the evaluation of myocardial viability. It can identify and quantify the extent of the scar tissue, myocardial perfusion, wall thickness and segmental wall motion, LV ejection fraction, ventricular volumes and contractile reserve with dobutamine stress [41]. It is also a useful method for evaluating myocardial viability, especially in patients with a poor ultrasonic window. It can precisely evaluate wall thickness and contractile function. With this technique, LV wall segments with diminished contractility and end-diastolic LV wall thickness over 5.5–6 mmm are considered viable. LV wall thickness less than 6 mm identifies scar with a sensitivity of 96% and specificity of 38% [62]. However, around 20% of the thinned and diminished contractility segments are viable with limited scar burden, and demonstrate contractility improvement and resolution of wall thinning after revascularization [24].

Membrane cellular damage and impaired micro perfusion can be detected with contrast-enhanced MRI. The most commonly used contrast agents are gadolinium-based, and are rapidly moving through the vascular space before entering to intercellular space. In heathy myocardial cells, gadolinium respects the intracellular space. The detection of myocardial perfusion defects using first pass gadolinium-enhanced MRI after an acute myocardial infarction indicates no-reflow and non-viable myocardium, and revascularization is not leading to the recovery of contractile function [4,63,64]. The normal myocardium has no gadolinium contrast retention, which is rapidly cleared after injection. Gadolinium enters and reaches high concentrations in the cellular space if the myocardial cells are irreversibly damaged and concentrates in fibrous scar tissue, as well. Late gadolinium-enhancement (LGE) cardiac MRI will produce a bright image in non-viable myocardium, due to the retained contrast, with a delayed clearance from an increased interstitial space [61]. The extent and the transmural extension of the myocardial scar can be accurately quantified due to superior spatial resolution. If transmurality on the LGE of a myocardial segment is greater than 50%, it is considered a non-viable myocardium, while if it is less than 50%, it is viable and correlates with LV segmental contractile restoration after revascularization [65,66]. 

Several clinical studies revealed the recovery of function in the viable ventricular wall detected by LGE [65,67,68]. The degree of contractile function recovery is proportional with the amount of viable transmural myocardium. There are five groups based on the probability of myocardial contractility recovery of the studied area: (a) myocardium without delayed enhancement (without fibrosis), with a probability of 80% for contractility recovery; (b) myocardium with 1–25% of the area of the segment with delayed enhancement has a 60% probability of recovery of contractility; (c) the group with a delayed enhancement of 26–50% of cardiac muscle has a reduced probability of contractile function restoring of 40%; (d) the group with 51–75% of compromise myocardial segment with only 10% of cases improving contractility; and (e) the group with more than 75% of the area of the myocardial segment affected has a probability of contractile function improvement of less than 1% [69].

Cardiac MRI is a simple, reproductible, and safe method, that has high accuracy for detecting viable myocardium. The good spatial resolution permits the detection of the extent of the viability alteration in the ventricular wall and clearly distinguishes between viable and non-viable myocardium [70]. Due to a better spatial resolution, LGE cardiac MRI can detect better subendocardial defects than PET and shows a good correlation with the perfusion–metabolism mismatch, the conventional gold standard for hibernating myocardium [71] or with SPECT [58]. 

In areas with LGE < 50% of segmental thickness, or with akinetic and thinned myocardium, the contractile reserve with a low dose of dobutamine will be evaluated [41]. A significant systolic thickening of 2 mm or more in segments with akinesia or dyskinesia during stress cardiac MRI can indicate viable myocardium [72]. Dobutamine stress cardiac MRI has a sensitivity of 81% and high specificity of 91%. By combining this high specificity method with the high sensitivity LGE, an increased accuracy in myocardial viability detection is obtained. A meta-analysis of 24 studies reported a sensitivity of LGE of 95% and a specificity of 91% for dobutamine stress cardiac MRI [62].

The volume fraction of the extracellular space can be evaluated based on native and post-contrast T1 images, a technique that can help in predicting myocardial function recovery [73,74]. A new cardiac MRI technique that is similar with speckle-tracking echocardiography can assess regional and longitudinal strain and can identify myocardial scar detected by LGE without the need for contrast administration and with a reduced scan time [75].

By combining multiple viability parameters, end-diastolic segmental thickness, LGE, and contractile reserve, an increased sensitivity and specificity are obtained in detecting viable myocardium (<50% LGE with contractile reserve), partial viable myocardium (non-transmural LGE or very thinned myocardium without contractile reserve), or non-viable myocardium (transmural scar) [41,76]. 

Any residual viable myocardium may benefit from revascularization, because even if there was no significant contractile recovery after revascularization, the improved outcome was observed in the surgical arm of the STICH trial [77].

The large-scale implementation of cardiac MRI for viability evaluation is limited, being an expensive and less available method, with a few contraindications (non-MRI conditional pacemakers and devices, metallic implants, and severe renal disease or unstable patients) [78].

#### 4.2.5. Cardiac Computed Tomography

In contrast, in enhanced cardiac computed tomography (CT), the contrast agent directly evaluates extracellular volume dimensions by interference with an X-ray beam, but due to a molecule with a reduced size of the iodine contrast dye, the optimal imaging time is shorter. Multi-row detector CT has greater spatial resolution and can accurately evaluate the myocardial and microvascular structure. Compared with LGE-cardiac MRI, the lower contrast-to-noise ratio represents a limitation. More extensive studies are necessary regarding the potential of multi-energy CT in myocardial viability assessment [12,79].

#### 4.2.6. Coronary Angiography

Coronarography and intravascular imaging can evaluate the morphology and function of the coronary vessels and the myocardium. Elements that are indicating a myocardium with preserved viability are: a distal TIMI (thrombolysis in myocardial infarction) grade III flow, myocardial blush of contrast, the presence of a collateral flow, and a significant proximal coronary stenosis. Myocardial contractility improvements after dobutamine or nitroglycerine also reflect myocardial viability. Coronary artery pressure and velocities can be measured invasively during adenosine-induced hyperemia. The fractional flow reserve during hyperemia is influenced by the severity of the coronary artery stenosis but also by the magnitude of the area of viable myocardium supplied by the stenotic coronary and the collateral vessels. The fractional flow reserve distal to a stenosis is lower in the presence of a viable myocardium, and the improvement after revascularization predicts myocardial contractility recovery (with a cutoff value of 0.70) [12,80].

In viable myocardium, the coronary microvascular bed is preserved and has normal resistance. Myocardial blood flow is reduced in infarcted myocardium due to microvascular dysfunction. The degree of microvascular impairment is a predictor of myocardial contractility recovery after revascularization in acute myocardial infarction. Several parameters can evaluate microvascular resistance as: coronary flow reserve, index of microcirculatory resistance, and hyperemic microvascular resistance. While coronary flow reserve is affected by the presence of epicardial coronary artery stenosis, the other two parameters reflect better microcirculatory dysfunction and are more specific for myocardial viability evaluation [81]. The index of microcirculatory resistance determined after revascularization of an acute myocardial infarction can predict the presence of viable myocardium and LV function recovery [82]. The improvement of coronary flow reserve after revascularization in patients with chronic ischemia is an indicator of myocardial function recovery. Other, less accurate, angiography parameters for coronary microcirculation evaluation are myocardial blush grade, TIMI grade flow, and TIMI frame count.

#### 4.2.7. Electrocardiography

Pathologic Q waves were traditionally attributed to transmural ischemia and thought to represent necrotic scarred myocardial tissue. On succeeding investigation, it was revealed that the presence of the Q wave has a poor correlation with the absence of myocardial viability, and low accuracy for myocardial viability detection compared with other imaging tests with a reduced sensitivity (65%) and specificity (69%) [83,84]. 

ST segment elevation in infarct-related leads and reciprocal ST depression during an exercise stress test have similar sensitivity and specificity for viability detection (84% and 100%) [85]. The normalization of abnormal T waves during exercise was showed in more recent studies to have poor sensitivity for myocardial viability detection [86,87].

There is no recommendation for a specific imaging modality for viability testing in the current guidelines [88], but CMR and PET should be considered when they are available due to an increased sensitivity and high spatial resolution, especially in patients with severely dilated and dysfunctional LV with thinned wall.

Different imaging modalities are reported to have different performance characteristics for the detection of myocardial viability. However, pooled data from a large number of patients enrolled in 105 studies revealed similar accuracy for SPECT, PET, and DSE, with higher sensitivity for PET and SPECT-Tl 201-rest-redistribution and a higher specificity for DES [89]. LGE cardiac MRI affecting > 50% of wall thickness has 90% negative predictive value for recovery of myocardial dysfunction, the highest compared to PET, SPECT, and DSE. The positive predictive value for LGE cardiac MRI is similar with PET and SPECT around 70–74%, but dobutamine cardiac MRI has the highest positive predictive value of 93%, and the lowest negative predictive value of 75% [12]. 

## 5. Myocardial Viability Related Studies

Viable myocardium can be identified by different imaging techniques, but its prognostic outcome after revascularization is still controversial.

Several studies have addressed this issue, mainly retrospective, observational studies with incomplete control of confounding factors. The results of these observational studies revealed improved LV function in patients with viable myocardium after revascularization and sustained the utility of myocardial viability assessment in clinical practice. Due to a non-randomized design, it is probably that the results at viability assessment determined the decision of revascularization, leading to important selection bias. Furthermore, there were important contemporary advancements in the medical treatment of heart failure and in the revascularization techniques, so the extrapolation of these data to contemporary medical practice is difficult [13].

Several observational studies revealed an improved outcome after revascularization in patients with ischemic heart failure and a significant amount of viable myocardium. The greater the area of viable myocardium, the better the result of revascularization. The optimal threshold for the area of viable myocardium is 25.8% for PET-FDG and 38.7% for SPECT [90]. A significant improvement of the global and segmental function of the LV [91,92], restoration of wall thickness, reverse remodeling of LV [91], and improved survival [93,94] after revascularization of viable myocardium were reported in these non-randomized studies.

Revascularization was compared to medical therapy in patients with severe CAD and LV dysfunction, evaluated for myocardial viability in a meta-analysis of 24 observational studies published by Allman et al. They followed-up with 3.088 patients, with LV ejection fraction (of 32 ± 8 %), during 25 ± 10 months. In patients with viable myocardium, revascularization was correlated with a 79.6% decrease in annual mortality, and there was a direct relationship between the severity of LV dysfunction and the importance of benefits after revascularization (*p* < 0.001). This meta-analysis concluded that testing for myocardial viability in patients with significant ischemic LV dysfunction can identify patients at increased risk, a risk that can be reduced with revascularization, with the magnitude of benefit increasing with the severity of LV dysfunction. This would be very important when deciding on revascularization in this challenging category of patients. Furthermore, patients with non-viable myocardium who underwent surgical revascularization had a higher mortality than patients with viable myocardium, meaning that for patients without myocardial viability, medical therapy is the preferred option [7].

One of the most important prospective observational studies was performed by Ling et al. on a number of 648 patients with ischemic cardiomyopathy, with a mean LV ejection fraction of 31%, with PET perfusion, and metabolic imaging testing. Early revascularization was accomplished in 33% of them, and they were followed-up for all-cause mortality as a primary endpoint. After a period of 2.8 years, the study observed a significant interaction between revascularization, the amount of hibernating myocardium, diabetes, and outcome (*p* < 0.0009). The risk of death increased in parallel with the amount of hibernating myocardium in patients who remained on medical therapy. At a level of 10% of hibernating myocardium, the benefits of medical and revascularization therapies were similar. Revascularization therapies had a progressively greater benefit as the amount of hibernating myocardium increased [95]. Another prospective observational study that used quantitative rest-redistribution of Thallium 201 scintigraphy to evaluate viability reported that patients with ischemic cardiomyopathy with poor viability had a higher rate of death or cardiac transplantation following CABG than patients with good viability [96].

The most important randomized controlled trials that addressed this issue are as follows: the PET and Recovery Following Revascularization (PARR-2) study, the Surgical Treatment for Ischemic Heart failure (STICH) study, and more recently, the REVIVED BCIS2 trial. 

The PARR-2 study was a randomized controlled trial that evaluated the role of viability testing on clinical outcomes. The trial enrolled patients with severe LV dysfunction and suspected CAD proposed for revascularization or transplantation and randomized to FDG-PET imaging (218 patients) or standard of care (212 patients). The primary endpoint was a composite of cardiac death, myocardial infarction, or recurrent hospitalization. At 1 year, it was registered in 30% of patients in the FDG-PET arm vs. 36% in the standard of care arm (RR 0.82, 95% CI 0.59–1.14, *p* = 0.16), suggesting no benefit for FDG-PET testing [97]. However, an important proportion of patients with high or moderate viability did not undergo revascularization, and this could generate an apparent absence of benefit associated with FDG-PET testing. In the subgroup of patients that followed the PET recommended strategy (76%), there was a significant improved outcome (HR = 0.62, 95% CI 0.42–0.93, *p* = 0.019). At 5 years of follow-up, there were still no significant differences between the two arms of study, but in the subgroup of patients that followed the PET-guided therapy, a significant reduction in the primary endpoint was registered (HR 0.73, 95% CI 0.54–0.99, *p* = 0.042) [98]. Approximatively 25% of patients in the PET-FDG arm did not follow the recommendations made based on PET imaging results, and 84% of them had either medium or high levels of viability. While no difference in mortality was observed between the PET-FDG and standard care groups, a decrease in rehospitalization due to cardiac causes was the major contributor to the composite endpoint favoring the PET-FDG group [98].

In the Ottawa-FIVE study, a post-hoc analysis from PARR-2, a significant benefit from PET guided management was revealed in an experienced heart failure center [99]. The outcome of patients evaluated for myocardial viability was better when FDG-PET was optimally used. The cardiac events were significantly reduced in the group with adherence to treatment recommendations based on PET-FDG results [98]. 

The Surgical Treatment for Ischemic Heart Failure (STICH) study was designed to investigate the role of CABG for the revascularization of patients with severe LV dysfunction [100]. This prospective randomized study compared 5-year outcomes in patients with severe ischemic LV dysfunction (LVEF < 35%) treated either with surgical revascularization combined with medical therapy including ACE-inhibitors, beta-blockers, statins, and diuretics or with medical therapy alone. A number of 1212 patients were enrolled in 99 centers with 610 patients in the group with CABG and optimal medical therapy (OMT) and 602 in the group with OMT alone. No difference in all-cause mortality was registered in the CABG group, with 36% vs. 41% in the OMT alone group (HR 0.86, 95% CI 0.72–1.04, *p* = 0.12). Cardiovascular death (28% CABG vs. 33% OMT, HR 0.81, 95% CI 0.66–1.00, *p* = 0.05) and cardiovascular death and hospitalization (68% OMT vs. 58% CABG, HR 0.74, 95% CI 0.64–0.85, *p* < 0.001) were significantly reduced in the CABG group. From 602 patients assigned in the OMT group, 17% crossed over to CABG before the end of the follow-up period. After excluding crossover, CABG was found to reduce significantly all-cause mortality (HR 0.76, 95% CI 0.62–0.92, *p* = 0.005). Failure to show a difference in all-cause mortality may also be explained by early mortality in the CABG group correlated with the surgical intervention. A sub-study from the STICH trial evaluated the role of viability testing and revealed no interaction between myocardial viability and outcomes. In total, 601 patients were tested for myocardial viability. The original protocol was that all the patients should undergo viability testing, but this was an impediment for recruitment, so the protocol was revised, and the myocardial viability assessment became optional, with either SPECT or DSE. SPECT myocardial perfusion imaging used a 17-segment model and viability was defined if >11 segments were viable based on relative tracer activity. For DSE, a 16-segment model was used, and viability was defined if >5 segments with hypo-akinesia at rest proved contractile reserve with dobutamine. From the patients who underwent a myocardial viability test, 487 patients were considered viable (49.9% underwent medical therapy and 50.1% underwent CABG), and 114 patients were considered non-viable (52.6% followed medical therapy and 47.4% underwent CABG) [101]. The viability sub-study from STICH was non-randomized, non-blinded, and underpowered. Imaging techniques SPECT and DSE used in STICH had limited power in differentiating between hibernating, subendocardial myocardium or transmural scar, in a thinned myocardial segment. Furthermore, a simple characterization of myocardium as viable or non-viable, with no description of various degrees of partial viable myocardium might have important implications on outcomes.

Myocardial viability was associated with a reduced overall rate of death in a univariate analysis (HR 0.64, 95% CI 0.48–0.86, *p* = 0.003), but in a multivariate model only a trend (although not statistically significant) to a reduced rate of mortality plus cardiovascular hospitalization was observed (55% CABG vs. 72.1% OMT, HR 0.55, 95% CI 0.55–0.85, *p* = 0.3903) [102]. The conclusion was that myocardial viability testing was not a predictor of outcome following revascularization; although, CABG was protective against cardiovascular death, an effect that persisted on extended follow-up [101]. 

A lot of explanations were found for these surprising results: (a) STICH included patients with angina rather than heart failure symptoms; (b) the benefit of viability testing was difficult to assess due to the reduced number of patients without viable myocardium (19%); (c) the baseline characteristics, medical, and interventional therapy in the viability group were different compared to the main trial population and were not representative for the ischemic cardiomyopathy population at large; (d) a high percentage of patients who underwent viability testing were with one vessel disease (25.3%), most probably patients with non-ischemic cardiomyopathy and incidental CAD, who were not expected to benefit from revascularization; and (e) a viability assessment was conducted with SPECT and DSE, at the discretion of the physicians, while in contemporary practice, these were replaced by PET and cardiac MRI. At 10 years of follow-up, the STICHES study found improved survival in the surgical arm, but again no interaction between myocardial viability and outcomes was proved, and furthermore better outcomes were independent of LV systolic function recovery [102]. 

The REVIVED-BCIS2 trial was the first study with randomized evidence on PCI for ischemic cardiomyopathy. It was a prospective multi-center, open label, randomized controlled trial that randomized 700 patients with severe CAD, LV ejection fraction < 35%, and myocardial viability in ≥4 dysfunctional segments to either PCI with complete revascularization encouraged or to medical therapy alone. The primary endpoint was a composite of all-cause mortality or hospitalization for heart failure and occurred in 37.2% in the PCI group and in 38% in the OMT group (HR = 0, 0.99; *p* =0.96) over a median period of 3.4 years follow-up. LV ejection fraction, which was 27.0% in both treatment groups at baseline, was no different between the two treatment approaches at 6 and 12 months. Quality of life favored the group with PCI at 6 and 12 months, but at 24 months no significant differences were seen [103,104]. Finally, the results of the REVIVED-BCIS2 trial revealed that interventional revascularization with PCI failed to provide any additional benefit beyond OMT in patients with severely impaired LV function and extensive CAD. The conclusion was that in patients with LV dysfunction, stable on OMT, PCI should not be recommended. However, revascularization should be considered in patients presenting with acute coronary syndromes or angina. The results of additional analyses and follow-up data from the REVIVED trial are awaited. Additionally, the concept of myocardial hibernation was challenged again with the results of the REVIVED BCIS trial, since only patients with demonstrable myocardial viability were enrolled [103,104].

The smaller Heart Failure Revascularization Trial (HEART) did not find a survival benefit in revascularized patients with ischemic heart failure versus conservative therapy. The HEART was terminated earlier due to poor recruitment; it was an underpowered study that showed no significant benefit in patients with ischemic heart failure, with LV ejection fraction ≤ 35%, and a significant proportion of viable myocardium who underwent revascularization compared to those on medical therapy alone [105].

Multiple studies proved better accuracy for LGE cardiac MRI in detecting myocardial viability, but the investigation was not available in PARR2 and STICH. Cardiac MRI is superior compared to SPECT and PET in detecting myocardial viability [106,107]. Kuhl et al. revealed that LGE cardiac MRI is more sensitive in predicting functional recovery than a combination of PET and SPECT (97% vs. 87%), and has a higher negative predictive value (93% vs. 77%) [108]. A prospective study evaluated the survival of 144 patients with CAD and severe LV dysfunction (mean ejection fraction: 24 ± 7%) who underwent myocardial viability assessment by LGE cardiac MRI. Medically treated patients with dysfunctional but viable myocardium had a significantly lower survival compared to patients with non-viable myocardium (48% vs. 77%, *p* = 0.02). Patients with severe LV dysfunction of ischemic etiology and viable myocardium detected by LGE cardiac MRI had significantly higher mortality without revascularization compared to those with complete revascularization (HR = 4.56, 95% CI: 1.93–10.8). The interaction between myocardial viability by LGE cardiac MRI and revascularization significantly predicted survival in ischemic heart failure with low ejection fraction [109]. 

In animal studies, it was demonstrated that the presence of viable non-revascularized myocardium was correlated with poor prognosis due to an increased risk of electrical instability leading to sudden cardiac death [110]. Hibernating myocardium is exposed to repeated episodes of ischemia and reperfusion leading to rhythm disturbances. Studies with LGE cardiac MRI revealed that in the peri-infarct zone, the necrosis is not transmural and viable cells coexist with scar tissue, an increased arrhythmogenic vulnerability being present [111,112].

Another small retrospective study was performed in a single center on patients with severe LV dysfunction (ejection fraction ≤ 35%), with a luminal narrowing of ≥50% in at least one major coronary artery, and myocardial viability tested using FDG-PET. Fifty-three patients underwent CABG and were divided in two groups: one with scar burden ≥ 10% (n = 30) and another with scar burden < 10% (n = 23). In patients with substantial scar amount, the presence of hibernating myocardium was followed by a functional recovery after revascularization, but this was not translated into survival benefit. The viability guided therapeutic strategy was not correlated with improved clinical outcome. The study confirmed that surgical coronary revascularization led to structural and functional improvement vs. medical therapy alone, but these improvements were independent of the baseline viability status. No benefits on survival, or major adverse cardiac and cerebrovascular events were observed between the groups with CABG vs. medical therapy alone. The authors concluded that, at least in part, this was explained by the high peri-operative mortality correlated with CABG, and possibly surgical revascularization is sometimes not enough to improve micro-circulation in the ischemic area, for the salvation of hibernating myocardium [113].

The results regarding the usefulness of myocardial viability tests remained inconclusive after analyzing thirty-two non-randomized (4328 patients) and four randomized (1079 patients) studies, in a systematic review and meta-analysis that compared medical treatment versus revascularization, in patients with viable and non-viable myocardium. Patients with viable myocardium but also without viable myocardium appeared to benefit from revascularization [114]. Another recent meta-analysis on 12 eligible studies that included a total of 1363 patients with ischemic cardiomyopathy and non-viable myocardium, of whom 501 patients underwent revascularization and 862 patients received medical therapy alone, found a significant reduction in all-cause mortality in the revascularization group, suggesting a benefit from revascularization compared to medical therapy, despite the absence of myocardial viability [115].

Results are awaited from AIMI-HF, an ongoing multi-center randomized trial and registry that evaluates the outcomes of patients with ischemic left ventricular dysfunction (EF < 45%) managed with advanced imaging methods (PET or cardiac MRI) compared to standard cardiac imaging (SPECT). The study is expected to enroll 1511 patients and follow them for at least 2 years. The composite primary outcome is cardiac death, myocardial infarction, resuscitated cardiac arrest, or cardiac hospitalization. A demonstration of viability detection is not mandatory for study enrollment, but its detection will influence the management and will be an important secondary analysis [116].

## 6. Hibernating Myocardium and Prognosis in Ischemic Heart Failure

### 6.1. Revascularization and Prognosis

The outcome after revascularization of hibernating myocardium is still an area of debate since the results of the randomized clinical trials are not supporting the findings from previous observational studies. Furthermore, the usefulness of viability testing prior to the revascularization procedure became unclear after publishing the results from the STICH trial and REVIVED BCSI2 [104,117]. 

However, the follow-up of patients from the STICH trial after 20 years of enrollment showed a trend for better survival in the group of patients with viability, compared to the non-viable group, and once again assessing myocardial viability came into focus [26]. Imaging tools such as SPECT and DSE used in STICH could miss some cases of myocardial viability, and patients were wrongly included in the non-viable group. PET and cardiac MRI can detect myocardial viability in cases where DSE and SPECT are failing, but these imaging tools were not available in STICH [77,97]. 

Yet, what is clear is that patients with severe LV remodeling, lower ejection fraction, larger LV volume, and more extensive CAD are more likely to benefit from surgical revascularization. These patients are at the highest risk for future acute coronary events, will tolerate less a new event, and will benefit more after CABG.

It seems that improvement of the LV function is not the only mechanism, and is not the most important mechanism for improved outcomes after CABG [77]. A relatively small improvement in LV ejection fraction was observed in the early stages following revascularization in STICH. The mean improvement in LV ejection fraction was 2% at 4 months in a group with an important amount of viable myocardium [18]. There are multiple factors involved in this: viable areas are not necessarily with contractile dysfunction, the regional nature of LV dysfunction that can result from various combinations of scarred and hibernating myocardium, the completeness and durability of revascularization, and the degree of peri-operative myocardial injury [118]. On the other side, after revascularization, sometimes a longer time of recovery may be necessary [119]. In the STICH viability sub-study, improvement of LVEF at 4 months was also observed in patients with optimization of medical treatment and was not correlated with long-term outcomes [77]. A small number of patients from STICH had a significant improvement (≥10%) of ejection fraction at 24 months after randomization [120] associated with reduced mortality. This improvement of the LV ejection fraction was not correlated with the type of treatment, and this further sustains that improving of LV function is not the most important mechanism that is leading to increased survival after CABG [121]. Beyond recovery of LV function, the most important beneficial mechanism for CABG is protection from future acute coronary events, and preventing of further damage and of sudden death [122]. Other mechanisms involved in the benefit of CABG are myocardial ischemia improvement due to improvement of coronary flow reserve and restoration of electrical stability of myocytes. CABG is improving survival, but also exercise tolerance and quality of life [123,124]. An important factor that should be considered is if there is concordance between the viable segments and the suitability for revascularization of the vessels that are distributing blood to that area. If there is concordance, CABG revascularization will protect the myocardium from future ischemic and arrhythmic events. In addition, this is more relevant in patients with multi-vessel disease and LV dysfunction [125]. 

The risk of death from heart failure in patients with ischemic LV dysfunction is clearly correlated to LV ejection fraction, but sudden arrhythmic or ischemic deaths are not. In the Digitalis Intervention Group trial, after analyzing the connection between LV ejection fraction and mechanism of death in 7788 patients (69% of them with ischemic heart failure), a near-linear relationship was demonstrated between LV ejection fraction and death from worsening heart failure, but the risk of death due to arrhythmic events was constant across the spectrum of reduced LV ejection fraction [126]. Sudden death risk due to ventricular arrhythmic events and myocardial infarction [122,127] can be influenced by revascularization, independent of any effect on contractile function. Myocardial scar can produce electrical instability, but the relationship between the amount of scar and risk is non-linear [128,129]. The benefits of CABG in STICH probably resulted from the reduction in sudden deaths due to fatal ventricular arrhythmias, and the prevention of acute myocardial infarction. The border area between the infarcted and viable myocardium is characterized by a non-uniform conduction and varying refractoriness and has an important arrhythmogenic potential [130,131]. Arrhythmogenesis is further increased by ischemia via intracellular acidosis and membrane depolarization. Incomplete revascularization with residual ischemia and peri-procedural myocardial infarction can appear [118], and therefore the reduction of arrhythmic risk cannot be predicted precisely. The electrophysiological properties of the myocardium are influenced earlier and independent from LV function recovery after revascularization [132,133].

Reduction in LV volume and sphericity after revascularization are also important prognostic factors beyond the improvement of regional wall thickening and of LV ejection fraction [8,134].

The risk associated with CABG will determine the final decision of surgical revascularization. PCI has a lower procedural risk, but the benefits of interventional revascularization in patients with ischemic cardiomyopathy need to be further evaluated. No improvement in regional LV function was observed in the randomized studies of chronic total occlusion PCI, even in the presence of viable myocardium; however, these were not addressed to patients with severe LV systolic dysfunction [135,136]. More recently, the REVIVED-BCIS2 trial showed no additional benefit for PCI revascularization versus OMT alone, in patients with severely impaired LV function and extensive CAD [104].

Furthermore, the presence of extensive viability has been shown to predict the response to various treatment methods, such as pharmacological [137,138], and cardiac resynchronization therapy [139], and not only to revascularization. 

The available data suggest that revascularization is reducing mortality in patients with ischemic heart failure that have a substantial amount of viable myocardium. The benefits of revascularization in patients without a substantial amount of viable myocardium remain unclear. The treatment effect of CABG in STICH was independent of viability status, with similar reductions in sudden cardiac death and heart failure. It is probably appropriate to recommend CABG in patients with ischemic cardiomyopathy based on the extent of their CAD, rather than on the viability testing [17]. Furthermore, no imaging method can assess viability with perfect precision, and variable accuracy is described for different imaging tests, so the HEART Team can decide on revascularization if it is clinically indicated [140]. 

The relationship between myocardial viability and revascularization is further studied in the ongoing trial IMAGE-HF (Imaging Modalities to Assist with Guiding and Evaluation of patients with Heart Failure): a prospective study that will compare the effect of an advanced imaging technique such as PET and cardiac MRI on clinical outcomes of patients with ischemic heart failure compared to those using standard care, including SPECT [116].

### 6.2. Interaction between Myocardial Ischemia and Viability 

There are still many unknown elements regarding the interaction between ischemia and viability in predicting both clinical outcomes and treatment effects.

Myocardial hibernation is induced by recurrent episodes of inducible ischemia, and the reversal of hibernation depends on two factors: resolution of ischemia and a viable myocardium that is able to recover. The recovery of dysfunctional myocardium is most likely to occur in territories with both inducible ischemia and viability. 

There are a few observational data that support these findings, respectively, a greater diagnostic accuracy if both contractile reserve and ischemia are demonstrated via a biphasic response on DSE, versus the demonstration of contractile reserve alone [92,110]. In addition, in the CHRISTMAS trial, a more important improvement in the LV ejection fraction was observed in patients with a larger area of hibernating myocardium or hibernating and ischemic myocardium, suggesting that the pharmacological treatment of ischemic or hibernating myocardium is contributing to the improvement of LV function [135].

Patients with ischemic LV dysfunction have, in general, a multi-vessel complex CAD, so probably they have a large ischemic burden on functional testing. Hibernating myocardium itself is an adaptive response to ischemia, but the ability to demonstrate inducible ischemic contractile dysfunction may be limited, and it is difficult to evaluate ischemia when the myocardium is thinned or partially scarred [141].

Patients with ischemic LV dysfunction and important inducible ischemia have poorer outcomes. The benefits of revascularization are greater in those with more extensive ischemia in observational studies [142]. However, it is difficult to evaluate the treatment effect from observational data, since the revascularization decision is commonly influenced by comorbidities or the frailty of patients that increase the procedural risk or reduce the chance of benefit, despite the presence of extensive ischemia. 

The role of ischemia in selecting patients with ischemic LV dysfunction for revascularization is controversial. Observational studies observed that low-dose DSE allowed the risk stratification of outcome after revascularization [143]. Some studies have identified an association between the presence of inducible ischemia and better outcomes after revascularization, while others have shown that this becomes irrelevant when other clinical factors, such as scar burden and non-viable myocardium are considered [95,144]. Rizzello et al. identified that in patients already scheduled to undergo revascularization, viability was a strong predictor of long-term prognosis, but the magnitude of ischemia on DSE was not a predictor of cardiac mortality [92]. 

A sub-study of the ISCHEMIA trial evaluated the relation between inducible ischemia and the effect of revascularization on clinical outcomes, in patients with heart failure and a mid-range ejection fraction, and revealed improved results with revascularization vs. pharmacological treatment [145]. The value of this analysis is limited by a small sample size, interactions between ischemia and viability were not explored, and patients with severely reduced ejection fraction were excluded from the study [146]. 

In clinical practice, most of the patients undergo coronarography as an initial investigation, followed directly by angioplasty or they are sent to CABG, so the majority of them are not evaluated regarding myocardial viability. If the evidence for the importance of viability testing will increase, then development of viability tests that can be used as adjunctive to coronarography will improve the management of these patients [18].

### 6.3. Pharmacological Therapy and Prognosis in Ischemic Heart Failure

Pharmacological therapy can improve the balance between myocardial oxygen consumption and demand, can diminish myocardial ischemia and restore contractile function, and can reduce the risk of recurrent ischemic and arrhythmic events. Beta-blockers are reducing heart rate and myocardial oxygen consumption; increasing diastole and improving coronary perfusion; and decreasing the risk of arrhythmia, recurrent myocardial infarction, and sudden death [147]. In the CHRISTMAS trial, the effectiveness of beta-blockers in improving LV function was related to the extent of myocardial hibernation [147]. 

Angiotensin converting enzyme inhibitors (ACE), angiotensin receptor blockers, and aldosterone receptor antagonists are reducing LV end-diastolic volume, LV pressure, and wall stress, and are improving subendocardial flow. These drugs can reduce mortality and the risk of heart failure hospitalization and improve symptoms [148]. 

Angiotensin receptor-neprilysin inhibitor and sodium-glucose cotransporter-2 inhibitors reduce the worsening of heart failure, cardiovascular and all-cause mortality, and improve heart failure symptoms and quality of life [149,150,151]. 

Obviously, the pharmacological treatment has a key role in the management of patients with ischemic heart failure and should not be neglected even if patients are referred for revascularization. On the other hand, there is an interaction between pharmacological therapy, revascularization, and viability, so controlling for their individual effect is very difficult in clinical trials. An experimental approach to the treatment of hibernating myocardium is to consider regenerative techniques by injecting stem cells (mesenchymal stem cells and mononuclear cells) into models of myocardial ischemia, that can lead to the improvement of myocardial contractility [117].

Randomized trials are needed, which consider the scope of viability testing more broadly, not simply if there is viable myocardium or not, but also to evaluate the amount or the percentage of the myocardium that is viable and to determine a relation between the amount of viable but dysfunctional myocardium and the response to revascularization therapy. 

## 7. Clinical Approach and Management of Patients with Ischemic Heart Failure

There is a persisting debate regarding myocardial viability testing due to the controversial results observed in prospective randomized studies. Viability assessments continue to have a role in several clinical circumstances but should always be just one of the factors implied in the decision of revascularization. The severity of LV adverse remodeling and dysfunction, the probability of successful revascularization, the anatomical characteristics of the coronary arterial bed, comorbidities, and surgical or interventional risk should be taken into account as well. 

Patients with ischemic cardiomyopathy and reduced ejection fraction are at a high risk of future cardiovascular events. When myocardial viability testing reveals the presence of viable myocardium, an improved outcome is expected by the coronary revascularization of the anatomically corresponding coronary artery [7,100,101]. To obtain a significant improvement of >5% in LV ejection fraction after coronary revascularization, a hibernating myocardium of around 20% of LV mass is required [17]. On the other hand, when the scar is involving > 20% of LV myocardium or the number of scared segments is >4, the recovery of LV function after revascularization is less likely [152]. Restoration of contractile function cannot characterize entirely the benefits achieved after revascularization. Improving of functional class and symptoms, reduction of rhythm disturbances, and quality life improvement need also to be considered [12].

Viability testing imaging tools are evaluating different aspects of the myocardium, but it is obvious that viable myocardium cannot be categorized in a dichotomized fashion, and imaging modalities must reveal the physiologic complexities of viable and ischemic myocardium [17,55].

Current ESC guidelines recommend a class IIb level of evidence B for non-invasive stress imaging (cardiac MRI, DSE, SPECT, PET) for the evaluation of myocardial viability in patients with CAD, suitable for revascularization [148]. ACC AHA guidelines consider that “myocardial viability is used as one of the tools to inform decisions regarding revascularization in patients with high surgical risk or with complex medical problems” [153].

In patients with ischemic heart failure with a LV ejection fraction moderately or severely reduced, with significant perfusion defects, but without moderate or severe ischemia, with multiple important comorbidities and/or poor coronary anatomy, viability testing is required. In patients with severe angina, with significant ischemia, with critical left main CAD and good coronary anatomy, with normal or mildly reduced ejection fraction, and no comorbidities, viability testing is not needed [78]. 

The type of imaging test will be selected based on availability, local experience, specific advantages of each method, and clinical setting [154]. In patients with severe LV dysfunction, we should consider SPECT, FDG PET, or LGE cardiac MRI, i.e., methods with increased sensitivity. When results are equivocal on another test, we should consider FDG-PET or cardiac MRI whenever it is possible. When SPECT or DSE characterize myocardium as non-viable but there are clinical or paraclinical parameters that raise the suspicion of partially viable myocardium, a more advanced imaging technique such as cardiac MRI to differentiate between non-viable and partial viable myocardium is warranted [102,155]. Furthermore, with cardiac MRI, an accurate evaluation of the degree of LV remodeling and dysfunction is accomplished [17]. Combining the results of different imaging modalities can lead to a better management and more precise evaluation of viable myocardium [156]. If no advanced imaging (such as cardiac MRI) is available, the therapeutic approach should be based on a Heart Team decision, after a case-to-case analysis that will take into account multiple factors (myocardial viability and ischemia, LV remodeling and dysfunction, coexisting mitral and tricuspid functional regurgitation, feasibility for other interventions as cardiac resynchronization therapy, symptoms, frailty, comorbidities, procedural risk versus benefits, etc.). 

When the patients have left main CAD or severe proximal three-vessel disease, we should avoid DSE, and in patients with renal failure or implanted metallic devices, we should avoid LGE cardiac MRI.

A patient with angina, with myocardial ischemia, with partial or viable myocardium on echocardiography or SPECT with low-intermediate procedural risk will benefit from coronary revascularization. However, a patient with severe heart failure, with severely dilated and remodeled LV, with akinetic and thinned myocardium on echocardiography and absent of radiotracer uptake at SPECT, with Q waves on ECG, and without angina will have a better outcome with medical and device therapy.

A dysfunctional myocardium is a continuum starting from early hibernation with minimal remodeling and no scar, to an intermediate stage of partial viable thinned myocardium, and at the end myocardial scar and fibrosis [29]. If the procedural risk is acceptable, viable or partially viable myocardium should be considered for coronary revascularization. It may take several months or years to see a functional recovery after coronary revascularization in severely dilated and dysfunctional LV with viable or partial viable myocardium [157]. In patients with remodeled LV with large areas of scar, outcomes are not likely to improve with revascularization, and the procedural risk will outweigh the benefits [102]. If the myocardium is characterized by advanced imaging, such as cardiac MRI and PET, as non-viable, revascularization of a scarred segment without metabolic activity is not warranted because it probably will not improve outcomes [91,148,158].

**Figure 4:**
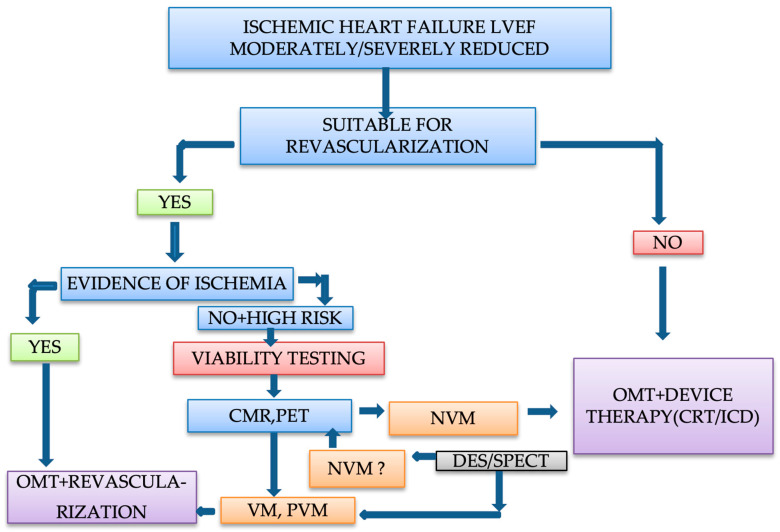
Management of patients with ischemic heart failure with moderately/severely reduced ejection fraction. Legend: LVEF—left ventricular ejection fraction; CMR—cardiac magnetic resonance; PET—positron emission tomography; DES—dobutamine stress echocardiography; SPECT—single-photon emission computerized tomography; OMT—optimal medical therapy; CRT—cardiac resynchronization therapy; ICD—internal cardioverter defibrillator; NVM—non-viable myocardium; VM—viable myocardium; PVM—partial viable myocardium.

The following clinical approach can be considered for the management of patients with ischemic heart failure with moderately or severely reduced ejection fraction (Figure 4).

When choosing between PCI or CABG, CABG is preferred in patients with multi-vessel complex CAD and in patients with diabetes due to improved outcomes and survival [152,157].

European revascularization guidelines have as a class I level of evidence B the recommendation for CABG in patients with ischemic cardiomyopathy and multi-vessel disease [88]. PCI can be recommended if there is the probability of complete revascularization in one- or two-vessel disease [159], and less in three-vessel disease after debating by the Heart Team (class II a, level of evidence C). In ACC-AHA guidelines, CABG is recommended in patients with ischemic cardiomyopathy, and no recommendations are provided regarding the use of PCI [160]. 

Heart failure guidelines also sustain guideline directed therapy and CABG in selected patients with LV ejection fractions of ≤35% [148,160].

All these recommendations are based on consensus opinion and the results from the STICHES extension study that revealed an improved survival at 10 years in patients with CABG plus OMT versus OMT alone [158,161]. When comparing long-term outcomes in patients undergoing revascularization by PCI or CABG in a retrospective cohort study on patients with LV ejection fraction < 35% and left anterior descending, left main or multi-vessel CAD, higher rates of mortality and major cardiac events were observed in patients undergoing PCI. The resulting conclusion was that CABG should be considered for most patients with severely reduced LV function suitable for revascularization [162]. However, in ischemic cardiomyopathy, the mortality rate remains very high: approximatively 20% at 3 years and 35% at 5 years [158].

Regarding interventional revascularization, after the first results of the REVIVED BCIS trial, no additional benefit over OMT was demonstrated in patients with severe CAD impaired LV function and viable myocardium [103,104].

However, the optimization of medical therapy should always play a key role in the management of all these patients because for medical therapy there is strong evidence that it is working. OMT can improve contractile function in viable segments independent of revascularization. 

## 8. Conclusions

Myocardial viability testing in the management of ischemic LV dysfunction remains controversial. Although multiple imaging tests can detect viable myocardium, cardiac MRI and PET whenever available should be preferred, due to their increased sensitivity and higher spatial resolution. Viability imaging continues to play a role in the highest risk patients with ischemic cardiomyopathy. Patients with very low ejection fraction, with three-vessel disease amenable for revascularization, large LV end-systolic volume, and ischemic mitral regurgitation may benefit from viability testing. CABG will lead to better outcomes in patients with large areas of hibernating myocardium, but when selecting a patient for revascularization, multiple factors should be considered. Patients with ischemic cardiomyopathy, with large areas of non-viable myocardium, may have better outcomes with medical therapy combined with device therapies. It remains unclear if patients without a certain amount of viable myocardium have no benefits after surgical revascularization. Further prospective trials of revascularization in patients with ischemic cardiomyopathy, with different amounts of viable myocardium, are needed to better define the value of identifying myocardial viability.

## Data Availability

Not applicable.
